# Bias, precision and statistical power of analysis of covariance in the analysis of randomized trials with baseline imbalance: a simulation study

**DOI:** 10.1186/1471-2288-14-49

**Published:** 2014-04-09

**Authors:** Bolaji E Egbewale, Martyn Lewis, Julius Sim

**Affiliations:** 1 Research Institute for Primary Care and Health Sciences, Keele University, ST5 5BG Staffordshire, UK

**Keywords:** Statistical analysis, Randomized controlled trials, Baseline imbalance, Bias, Precision, Statistical power

## Abstract

**Background:**

Analysis of variance (ANOVA), change-score analysis (CSA) and analysis of covariance (ANCOVA) respond differently to baseline imbalance in randomized controlled trials. However, no empirical studies appear to have quantified the differential bias and precision of estimates derived from these methods of analysis, and their relative statistical power, in relation to combinations of levels of key trial characteristics. This simulation study therefore examined the relative bias, precision and statistical power of these three analyses using simulated trial data.

**Methods:**

126 hypothetical trial scenarios were evaluated (126 000 datasets), each with continuous data simulated by using a combination of levels of: treatment effect; pretest-posttest correlation; direction and magnitude of baseline imbalance. The bias, precision and power of each method of analysis were calculated for each scenario.

**Results:**

Compared to the unbiased estimates produced by ANCOVA, both ANOVA and CSA are subject to bias, in relation to pretest-posttest correlation and the direction of baseline imbalance. Additionally, ANOVA and CSA are less precise than ANCOVA, especially when pretest-posttest correlation ≥ 0.3. When groups are balanced at baseline, ANCOVA is at least as powerful as the other analyses. Apparently greater power of ANOVA and CSA at certain imbalances is achieved in respect of a biased treatment effect.

**Conclusions:**

Across a range of correlations between pre- and post-treatment scores and at varying levels and direction of baseline imbalance, ANCOVA remains the optimum statistical method for the analysis of continuous outcomes in RCTs, in terms of bias, precision and statistical power.

## Background

Many randomized controlled trials (RCTs) involve a single post-treatment measurement of a continuous outcome variable previously measured at baseline. Although randomization creates asymptotic balance in important prognostic factors, including baseline values of the outcome variable [1], in finite samples an imbalance in such factors may occur notwithstanding randomization [2-6]; this represents the difference between the *expectation* of a random process and its *realization*[6]. Depending crucially on the correlation between the baseline covariate and the outcome variable, this chance imbalance may not only create a potential bias in crude estimates of treatment effect in the outcome variable, but may also affect the precision with which such an effect is measured and the statistical power of the analysis. Attempts are made to address this problem either at the level of design (e.g. stratification and minimization) or at the level of analysis, or indeed both. Although opinions are still divided on the first-line strategy to deal with baseline imbalance in RCTs [7-11], the general consensus seems to be that, whichever method is employed at the design stage to achieve balance in covariate distribution, an adjusted statistical analysis that accounts for important covariates should take precedence over an unadjusted analysis [3,8,9,12-16]. Nonetheless, there appears to be varied practice in this area and further consideration of the relative merits of adjusted and unadjusted analyses has been called for [17].

For a single post-treatment assessment of a continuous outcome variable, three statistical methods have commonly been used: crude comparison of treatment effect by *t* test or, equivalently, analysis of variance (ANOVA); change-score analysis (CSA); and analysis of covariance (ANCOVA). On occasions, CSA is performed using percentage change, but this has been shown to be an inefficient approach [18]. Whereas CSA compares changes between pre- and post-treatment scores between treatment groups, ANCOVA accounts for the imbalance by including baseline values in a regression model – theoretically, this regression-based procedure yields unbiased estimates of treatment effect [19,20].

Given their different statistical basis, each of these statistical methods has a potentially marked effect on the estimate of the treatment effect and its associated precision, and differing statistical conclusions may therefore be reached according to the method of analysis chosen [21-23]. In addition, contrary views have been reported on the implications of using CSA as a method for statistical adjustment in an RCT [3,12,24,25] and this warrants further investigation, to clarify the appropriateness of particular methods.

This study therefore seeks to quantify, through an established approach based on data simulation [22,26-28], differences in the estimate (bias) and precision of treatment effect and associated statistical power through using either ANOVA or CSA in relation to the unbiased estimate of treatment effect by ANCOVA, in differing hypothetical trial scenarios. Although previous authors [19,29] have provided theoretical accounts of bias and precision in estimates of treatment effect derived through ANOVA and CSA when baseline imbalance exists, we are aware of no previous study that has sought simultaneously to quantify bias, precision and statistical power of these three methods in relation to a wide range of combinations of different levels of experimental conditions, including baseline imbalance in the outcome variable, that are typical of pragmatic RCTs. Addressing this issue will allow practical recommendations to be made for the future analysis of RCTs in the presence of baseline imbalance.

## Methods

### Data simulation

A statistical program was developed in STATA to generate hypothetical two-arm trials involving specific levels of experimental conditions, run the regression models for the statistical methods being studied, and then post selected results into a file. Each hypothetical trial scenario was repeated a thousand times, so as to generate robust estimates (e.g. allowing statistical power to be estimated with a margin of error no greater than ±3% at a 95% confidence level). Detailed information on the statistical program is included in the Appendix.

### Levels of experimental conditions

A population standard deviation of 1 (*σ* = 1) for the outcome data was assumed in each trial and these data were normally distributed at baseline and at follow-up. A 1:1 allocation ratio was employed. Rather than choose arbitrary levels of other experimental conditions, these were selected in relation to specific criteria so as to reproduce conditions typical of an empirical trial scenario. Data for the outcome variable (*Y*_T,_*Y*_C_, for the treatment and control groups, respectively, with higher values taken to be clinically desirable) were simulated so as to produce a standardized treatment effect $\left(Y_{T}^{\prime }-Y_{C}^{\prime }\right)$:

$$Y_{T}^{\prime }-Y_{C}^{\prime }=\frac{Y_{T}-Y_{C}}{\mathrm{SD}\left(Y\right)}$$

A treatment effect was taken to be a higher (i.e. better) score in the treatment than in the control group, and was set at three levels of 0.2, 0.5 and 0.8, classified by Cohen [30] as ‘low’, ‘medium’, and ‘large’ respectively.

For a nominal statistical power of 80%, the required sample size was utilized for each of these standardized effect sizes: 394, 64 and 26 per group, respectively. The correlation between baseline values (*Z*_T_, *Z*_C_, for the treatment and control groups, respectively) and post-treatment values was varied from 0.1 to 0.9 in increments of 0.2, as it has been argued that the correlation between baseline covariates and outcome scores in RCTs may range between these values [31]. A correlation of zero was also included as a reference value.

For each hypothetical trial, imbalance in baseline values of the outcome measure was computed as a standardized score $\left(Z_{T}^{\prime }-Z_{C}^{\prime }\right)$, in terms of its standard error:

$$Z_{T}^{\prime }-Z_{C}^{\prime }=\frac{Z_{T}-Z_{C}}{2\sqrt{n}}\times z$$

Here, *z* is a standard normal deviate. In this way, realistic values of imbalance were derived in relation to the sample size, thus avoiding large absolute imbalance for large sample sizes that would contradict the principles of randomization. Imbalance was simulated in both the same direction (‘positive’ imbalance, where the treatment group has ‘better’ baseline scores than the control group) and the opposite direction (‘negative’ imbalance, where the control group has ‘better’ scores) in relation to the treatment effect. The predetermined levels of $Z_{T}^{\prime }-Z_{C}^{\prime }$ for this study were calculated in relation to standard normal deviates of ±1.28, ±1.64 and ±1.96, representing 20%, 10% and 5% two-tailed probabilities respectively of the standard normal distribution.

Hence, the various levels of imbalance had a predetermined probability of occurring, whatever the sample size and on whatever scale the covariate or outcome variable is scored.

In total, 126 scenarios representing hypothetical combinations of experimental conditions were simulated at 80% nominal power, comprising:

7 standardized baseline imbalances: −1.96; −1.64; −1.28; 0; 1.28; 1.64; 1.96

6 covariate-outcome (*ZY*) correlations: 0; 0.1; 0.3; 0.5; 0.7; 0.9

3 standardized treatment effect sizes: 0.2; 0.5; 0.8

Each scenario was analysed by each of the statistical methods. In the analyses, a binary variable represented group allocation, such that the estimate of the treatment effect in each simulated dataset was derived from the associated regression coefficient (*β*).

### Bias, precision and power

To quantify bias associated with the estimates of effect by ANOVA and CSA, the following indices were computed:

$$\;\mathrm{bia}s_{\mathrm{ANOVA}}=\beta_{\mathrm{ANCOVA}}-\beta_{\mathrm{ANOVA}}$$

$$\mathrm{bia}s_{\mathrm{CSA}}=\beta_{\mathrm{ANCOVA}}-\beta_{\mathrm{CSA}}$$

Bias was assessed not in relation to the nominal standardized treatment effect, as this effect is liable to be biased in the presence of confounding. Rather, bias was determined in relation to the adjusted estimate from ANCOVA, as this is known to provide the unbiased estimate of outcome, conditional upon the conditions represented by a given scenario.

In order to quantify the relative precision of the three methods of analysis, ratios of the resulting standard errors (design effects) were calculated:

$$\frac{SE_{\mathrm{ANCOVA}}}{SE_{\mathrm{ANOVA}}}\;\frac{SE_{\mathrm{CSA}}}{SE_{\mathrm{ANOVA}}}\;\frac{SE_{\mathrm{ANCOVA}}}{SE_{\mathrm{CSA}}}$$

Finally, the conditional statistical power of each of the three methods of analysis was calculated as the percentage of rejections of the null hypothesis in the 1000 simulations within each scenario; this was compared to the nominal power of 80%.

## Results

### Bias

**Figure 1:**
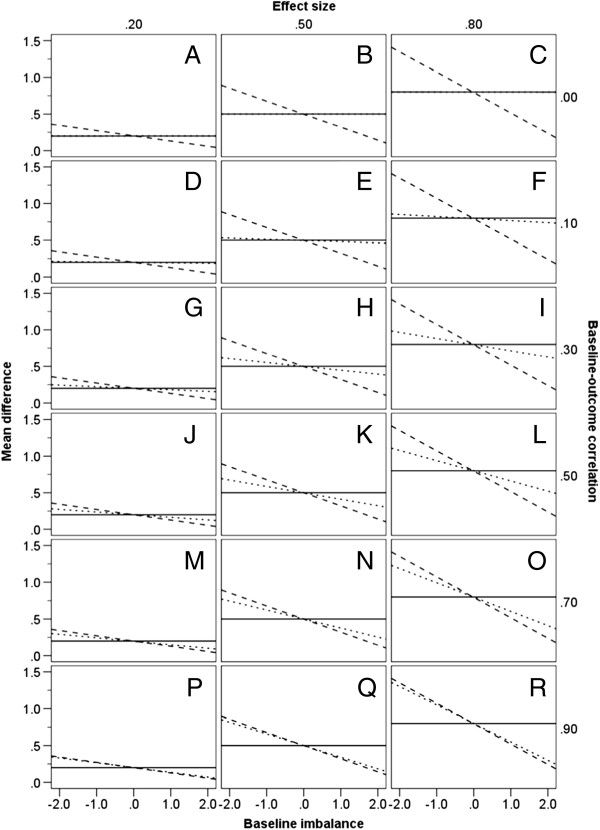
**Directional bias of statistical methods.** Estimates are given at differing levels of baseline-outcome correlation, treatment effect sizes, and baseline imbalance (−1.96, −1.64, −1.28, 0, 1.28, 1.64, 1.96). Estimates derived from ANCOVA represent the unbiased treatment effect. ANOVA – solid line; ANCOVA – dotted line; CSA – dashed line.

Figure 1 shows the mean estimated treatment effect and thereby the directional pattern of bias for ANOVA and CSA, in relation to ANCOVA as the reference unbiased analysis. Table 1 indicates the bias, in standardized (SD) units, for each of ANOVA and CSA, again in relation to ANCOVA. Values are given in the table conditional on the three treatment effects, the six levels of *ZY* correlation, the situation in which there is no baseline imbalance, and the six values of standardized imbalance.

**Table 1:** Bias (standard deviation units) in respect of ANCOVA versus ANOVA and ANCOVA versus CSA

**Difference**	***Z′***_***T***_ ***–Z′***_***C***_	**Treatment effect**																	
		**0.2**						**0.5**						**0.8**					
		***ZY *** **correlation**						***ZY *** **correlation**						***ZY *** **correlation**					
		**0**	**0.1**	**0.3**	**0.5**	**0.7**	**0.9**	**0**	**0.1**	**0.3**	**0.5**	**0.7**	**0.9**	**0**	**0.1**	**0.3**	**0.5**	**0.7**	**0.9**
ANCOVA – ANOVA	−1.96	0.00	0.01	0.04	0.07	0.10	0.13	0.00	0.03	0.10	0.17	0.24	0.31	0.00	0.05	0.16	0.27	0.38	0.49
	−1.64	0.00	0.01	0.04	0.06	0.08	0.11	0.00	0.03	0.09	0.14	0.20	0.26	0.00	0.04	0.14	0.23	0.31	0.41
	−1.28	0.00	0.01	0.03	0.05	0.06	0.08	0.00	0.02	0.07	0.11	0.16	0.20	0.00	0.03	0.10	0.17	0.25	0.32
	0.00	0.00	0.00	0.00	0.00	0.00	0.00	0.00	0.00	0.00	0.00	0.00	0.00	0.00	0.00	0.01	0.01	0.00	0.00
	1.28	0.00	−0.01	−0.03	−0.04	−0.06	−0.08	0.00	−0.03	−0.07	−0.11	−0.16	−0.21	0.00	−0.04	−0.11	−0.18	−0.25	−0.32
	1.64	0.00	−0.01	−0.03	−0.06	−0.08	−0.11	0.00	−0.03	−0.09	−0.15	−0.21	−0.26	0.00	−0.05	−0.14	−0.23	−0.32	−0.41
	1.96	0.00	−0.01	−0.04	−0.07	−0.10	−0.13	0.00	−0.04	−0.11	−0.18	−0.24	−0.31	0.00	−0.06	−0.17	−0.28	−0.38	−0.49
ANCOVA – CSA	−1.96	−0.14	−0.13	−0.10	−0.07	−0.04	−0.01	−0.35	−0.31	−0.24	−0.17	−0.10	−0.03	−0.55	−0.49	−0.38	−0.27	−0.16	−0.05
	−1.64	−0.12	−0.11	−0.08	−0.06	−0.04	−0.01	−0.29	−0.26	−0.20	−0.15	−0.09	−0.03	−0.46	−0.41	−0.31	−0.22	−0.14	−0.05
	−1.28	−0.09	−0.08	−0.06	−0.05	−0.03	−0.01	−0.23	−0.20	−0.16	−0.12	−0.07	−0.03	−0.36	−0.32	−0.25	−0.17	−0.10	−0.03
	0.00	0.00	0.00	0.00	0.00	0.00	0.00	0.00	0.00	0.00	0.00	0.00	0.00	0.00	0.00	0.00	0.00	0.00	0.00
	1.28	0.09	0.08	0.06	0.05	0.03	0.01	0.23	0.20	0.16	0.11	0.07	0.02	0.35	0.32	0.25	0.18	0.11	0.04
	1.64	0.12	0.10	0.08	0.06	0.04	0.01	0.29	0.26	0.20	0.15	0.09	0.03	0.45	0.41	0.32	0.23	0.14	0.05
	1.96	0.14	0.13	0.10	0.07	0.04	0.02	0.35	0.31	0.24	0.17	0.10	0.04	0.54	0.49	0.38	0.27	0.16	0.05

The results displayed in Figure 1 demonstrate that, when there is no imbalance at baseline (i.e. $Z_{T}^{\prime }-Z_{C}^{\prime }=0$), all three statistical methods yield the same unbiased estimate of treatment effect, irrespective of the level of *ZY* correlation or the standardized effect size. It is also clear that, for a given nominal treatment effect, the estimates yielded by ANOVA and CSA do not change in relation to the level of *ZY* correlation.

However, when treatment groups differ at baseline (i.e. $Z_{T}^{\prime }-Z_{C}^{\prime }\neq 0$) there is a noticeable difference in the estimate of treatment effect by these methods. The magnitude of this difference depends on the degree of *ZY* correlation and the size of baseline imbalance. At a given level of baseline imbalance, ANOVA and ANCOVA give precisely equivalent estimates when *ZY* correlation is zero (Figure 1 graphs A, B and C). However, the bias of ANOVA (relative to the unbiased estimates derived through ANCOVA) increases as *ZY* correlation rises and, holding *ZY* correlation constant, also increases with a higher degree of baseline imbalance. ANOVA and ANCOVA produce similar estimates of effect when *ZY* correlation is less than 0.3 (see, for example, Figure 1 graphs D, E and F), but at higher *ZY* correlations, the difference in the estimate of effect for the two methods becomes more obvious (see, for example, Figure 1 graphs M, N and O). This bias is equal in magnitude for either direction of imbalance. Thus, Table 1 shows there is a bias of 0.07 SD and −0.07 SD respectively associated with the estimate of effect by ANOVA when a standardized baseline imbalance of 1.96 exists in the same direction (i.e. $Z_{T}^{\prime }-Z_{C}^{\prime }>0$), or opposite direction (i.e. $Z_{T}^{\prime }-Z_{C}^{\prime }<0$), at a standardized treatment effect of 0.2 and a *ZY* correlation of 0.5 (see Figure 1 graph J).

If the *ZY* correlation is large, even a small imbalance yields a substantial bias in the estimate of treatment effect when using ANOVA (for example, Figure 1 graphs N and O). Conversely, if the *ZY* correlation is small, only a small bias results even if the baseline imbalance is large (for example, Figure 1 graphs H and I). Thus, from Table 1, when the ZY correlation is 0.7, ANOVA shows an upward bias with regard to ANCOVA of 0.25 SD at a standardized baseline imbalance of −1.28 and standardized treatment effect of 0.8. In contrast, when the ZY correlation is 0.3, a larger imbalance of −1.96 produces an upward bias for ANOVA of only 0.16 SD when estimating the same effect (Table 1).

Turning to CSA, the magnitude of bias similarly is greater with an increase in the absolute value of baseline imbalance, and is equal for both directions of baseline imbalance (see, for example, Figure 1 graphs K and L). It is apparent from Figure 1 and Table 1 that CSA produces an opposite bias to that induced by ANOVA; when the one method overestimates the unbiased treatment effect, the other method underestimates it, and vice versa. However, in contrast to the case of ANOVA, at a given level of baseline imbalance, bias in the estimate of effect through CSA decreases as *ZY* correlation increases. When baseline imbalance is in the same direction as the treatment effect (i.e. $Z_{T}^{\prime }-Z_{C}^{\prime }>0$), the estimate derived from CSA is markedly lower than that of either ANOVA or ANCOVA if *ZY* correlation is low (see, for example, Figure 1 graphs F and I). Here, CSA underestimates the true treatment effect to a much larger degree than ANOVA overestimates it. Conversely, the bias associated with CSA is much smaller than that of ANOVA if *ZY* correlation is high (see, for example, Figure 1 graphs O and R).

When *ZY* correlation is at or below 0.7, CSA yields the smallest estimate of treatment effect of the three methods if baseline imbalance is in the same direction $\left(Z_{T}^{\prime }-Z_{C}^{\prime }>0\right)$ as the treatment effect, and the largest estimate of effect if imbalance is in the opposite direction to the treatment effect $\left(Z_{T}^{\prime }-Z_{C}^{\prime }<0\right)$, indicating that it provides the strongest adjustment for baseline imbalance in these circumstances. The bias of ANOVA relative to ANCOVA can be expressed algebraically by the formula:

$$\left(Y_{T}^{\prime }-Y_{C}^{\prime }\right)\rho \frac{z}{-2\sqrt{\left|z\right|}},$$

and the bias of CSA to ANCOVA by the formula:

$$\left(Y_{T}^{\prime }-Y_{C}^{\prime }\right)\left(\rho -1\right)\frac{z}{-2\sqrt{\left|z\right|}}.$$

### Precision

**Figure 2:**
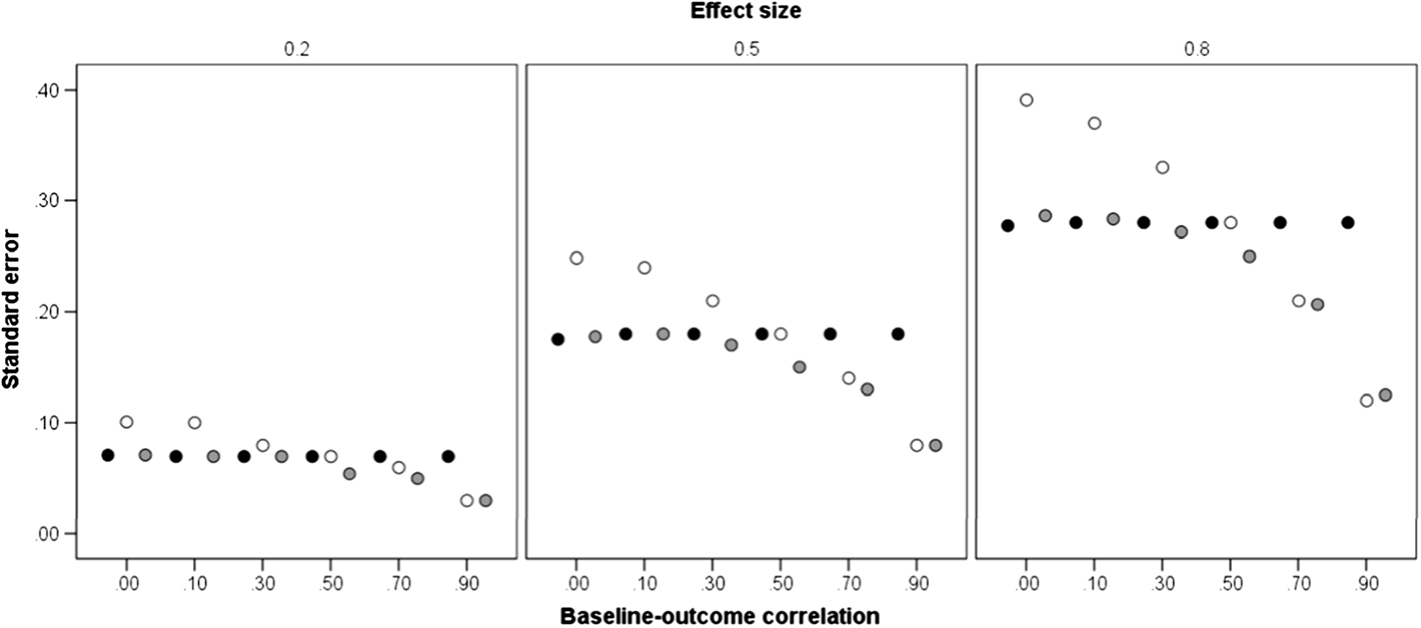
**Standard errors of statistical methods.** Estimates are given at differing levels of baseline-outcome correlation, conditional on treatment effect. The markers show the mean standard error, averaged across the treatment effects. ANOVA – black markers; ANCOVA – grey markers; CSA – white markers.

Figure 2 shows the mean standard error, at each standardized treatment effect size, for the three methods of analysis, at different levels of *ZY* correlation (the direction and magnitude of baseline imbalance was found to have no effect on precision and has therefore been ignored). The size of the standard error is proportional to the treatment effect, but this simply reflects the sample sizes corresponding to these effects. For ANOVA (black markers), the standard error is constant across *ZY* correlations, reflecting the fact that this analysis takes no account of the baseline values. For the other two analyses, it can be observed that the standard error associated with ANCOVA (grey markers) is similar to that of ANOVA at a low *ZY* correlation, but decreases monotonically as correlation increases. Standard errors for CSA (white markers) are, however, variable. At a low *ZY* correlation, mean standard error is markedly higher than that of both ANOVA and ANCOVA, whereas at *ZY* correlations above 0.5, it is markedly lower than that of ANOVA and comparable to that of ANCOVA. Overall, ANCOVA is the most precise analysis, especially at *ZY* correlations from 0.5 to 0.9.

Table 2 shows the relative precision of the three analyses, expressed as a ratio of their standard errors. As in Table 1, values of these ratios are given for the three treatment effects, the six levels of *ZY* correlation, the situation in which there is no baseline imbalance, and the six values of standardized imbalance. Ratios greater than unity indicate that the numerator analysis has a larger standard error (i.e. is less precise) than the denominator analysis. Table 2 confirms the equivalent precision of CSA and ANOVA at a correlation of 0.5. However, it shows that when *ZY* correlation is as low as 0.1, ANOVA can yield approximately a 36% gain in precision against CSA, whereas when *ZY* correlation is 0.9, CSA provides approximately a 57% gain in precision over ANOVA. Table 2 also indicates that only at a correlation of 0.7 or greater does CSA produce comparable precision to that of ANCOVA.

**Table 2:** Design effect (ratio of standard errors) in respect of ANCOVA versus ANOVA, CSA versus ANOVA, and ANCOVA versus CSA

**Ratio**	***Z′***_***T***_ ***–Z′***_***C***_							**Treatment effect**											
		**0.2**						**0.5**						**0.8**					
		***ZY *** **correlation**						***ZY *** **correlation**						***ZY *** **correlation**					
		**0**	**0.1**	**0.3**	**0.5**	**0.7**	**0.9**	**0**	**0.1**	**0.3**	**0.5**	**0.7**	**0.9**	**0**	**0.1**	**0.3**	**0.5**	**0.7**	**0.9**
ANCOVA/ANOVA	−1.96	1.00	1.01	0.97	0.86	0.71	0.43	1.02	1.01	0.97	0.88	0.72	0.44	1.05	1.04	1.00	0.90	0.75	0.45
	−1.64	1.00	1.01	0.97	0.87	0.73	0.44	1.01	1.01	0.96	0.88	0.72	0.44	1.04	1.02	0.99	0.90	0.74	0.45
	−1.28	1.00	1.00	0.97	0.87	0.73	0.43	1.01	1.01	0.96	0.87	0.73	0.44	1.03	1.02	0.98	0.89	0.73	0.45
	0.00	1.00	1.00	0.96	0.86	0.71	0.43	1.00	1.00	0.96	0.86	0.71	0.43	1.01	1.00	0.96	0.86	0.71	0.43
	1.28	1.00	1.00	0.97	0.86	0.71	0.43	1.01	1.03	0.93	0.83	0.70	0.43	1.03	1.01	0.97	0.89	0.72	0.45
	1.64	1.00	1.00	0.97	0.86	0.71	0.43	1.01	1.01	0.97	0.86	0.72	0.44	1.04	1.03	0.99	0.90	0.74	0.45
	1.96	1.00	1.00	0.97	0.86	0.71	0.43	1.02	1.00	0.97	0.86	0.72	0.44	1.05	1.04	1.00	0.90	0.75	0.46
CSA/ANOVA	−1.96	1.42	1.36	1.14	1.00	0.79	0.43	1.42	1.34	1.18	1.00	0.77	0.44	1.41	1.34	1.18	1.00	0.77	0.44
	−1.64	1.42	1.36	1.20	1.00	0.79	0.44	1.42	1.34	1.18	1.00	0.77	0.44	1.41	1.32	1.18	1.00	0.77	0.45
	−1.28	1.42	1.36	1.14	1.00	0.79	0.43	1.42	1.34	1.18	1.00	0.77	0.44	1.41	1.34	1.18	1.00	0.76	0.45
	0.00	1.42	1.36	1.14	1.00	0.79	0.43	1.42	1.34	1.19	1.00	0.77	0.44	1.41	1.34	1.18	1.00	0.77	0.45
	1.28	1.42	1.36	1.14	1.01	0.86	0.44	1.42	1.34	1.17	1.00	0.75	0.43	1.41	1.34	1.18	1.00	0.76	0.45
	1.64	1.42	1.37	1.20	1.00	0.79	0.43	1.42	1.34	1.18	1.00	0.77	0.44	1.41	1.34	1.18	1.00	0.76	0.45
	1.96	1.42	1.36	1.14	1.00	0.79	0.43	1.42	1.34	1.18	1.00	0.77	0.44	1.41	1.34	1.18	1.00	0.76	0.45
ANCOVA/CSA	−1.96	0.71	0.75	0.85	0.86	0.91	1.00	0.72	0.75	0.82	0.88	0.94	0.99	0.74	0.78	0.85	0.91	0.97	1.02
	−1.64	0.71	0.75	0.81	0.87	0.93	1.00	0.72	0.75	0.81	0.88	0.94	0.99	0.74	0.77	0.84	0.90	0.96	1.02
	−1.28	0.71	0.74	0.85	0.87	0.93	1.00	0.71	0.75	0.81	0.87	0.93	0.99	0.73	0.76	0.83	0.89	0.97	1.01
	0.00	0.71	0.74	0.84	0.86	0.91	1.00	0.71	0.74	0.81	0.87	0.93	0.97	0.72	0.75	0.81	0.87	0.93	0.99
	1.28	0.71	0.74	0.85	0.85	0.83	0.97	0.71	0.77	0.80	0.83	0.93	0.99	0.73	0.75	0.83	0.89	0.95	1.00
	1.64	0.71	0.73	0.81	0.86	0.91	1.00	0.72	0.75	0.82	0.86	0.94	0.99	0.73	0.77	0.83	0.90	0.98	1.02
	1.96	0.71	0.74	0.85	0.86	0.91	1.00	0.72	0.75	0.82	0.86	0.94	0.99	0.74	0.78	0.84	0.91	0.99	1.02

The computed ratio of the standard errors of ANCOVA and ANOVA from the simulated datasets approximately fits the algebraic expression $\sqrt{1-\rho^{2}}$, irrespective of whether or not treatment groups are balanced at baseline, and the ratios for CSA and ANOVA and for ANCOVA and CSA approximately fit the expressions $\sqrt{2\left(1-\rho \right)}$ and $\sqrt{\frac{\left(1-\rho^{2}\right)}{2\left(1-\rho \right)}},$ respectively.

### Statistical power

**Figure 3:**
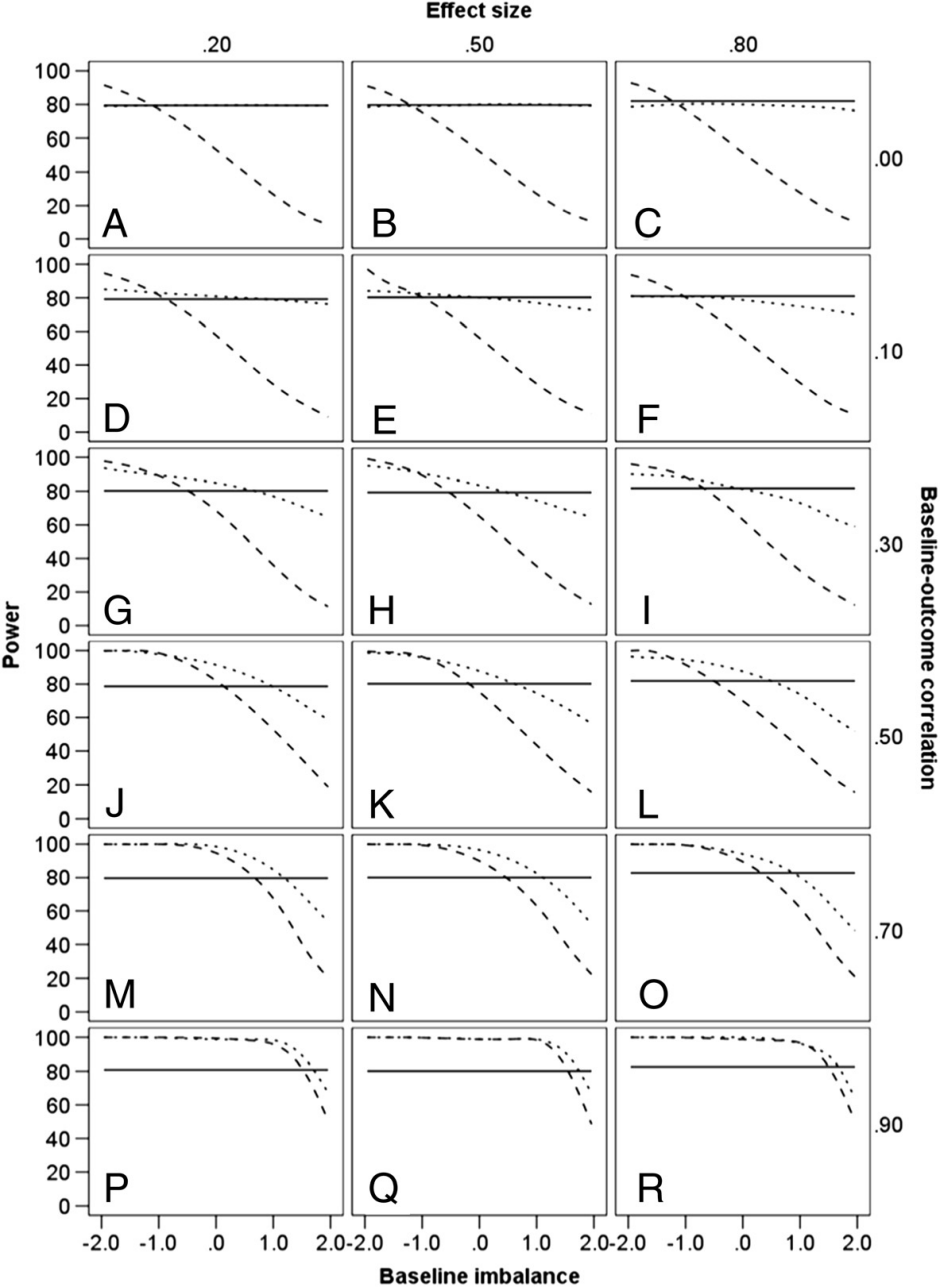
**Power (%) of statistical methods.** Estimates are given at differing levels of baseline-outcome correlation, treatment effect sizes, and baseline imbalance (−1.96, −1.64, −1.28, 0, 1.28, 1.64, 1.96). ANOVA – solid line; ANCOVA – dotted line; CSA – dashed line.

The power of ANCOVA, CSA and ANOVA is shown in Table 3 in terms of increments or decrements in relation to the nominal power of 80%, again conditional on treatment effect and levels of *ZY* correlation and baseline imbalance. Absolute values of power for ANCOVA, CSA and ANOVA are shown graphically in Figure 3.

**Table 3:** **Increments (positive values) and decrements (negative values) of power (%) for ANCOVA, ANOVA and CSA relative to a nominal power of 80% and conditional upon levels of baseline imbalance and *ZY *** **correlation**

	***Z′***_***T***_ ***–Z′***_***C***_	**Treatment effect**																	
		**0.2**						**0.5**						**0.8**					
		***ZY *** **correlation**						***ZY *** **correlation**						***ZY *** **correlation**					
**Analysis**		**0**	**0.1**	**0.3**	**0.5**	**0.7**	**0.9**	**0**	**0.1**	**0.3**	**0.5**	**0.7**	**0.9**	**0**	**0.1**	**0.3**	**0.5**	**0.7**	**0.9**
ANCOVA	−1.96	−1.00	5.10	13.60	>19.9	>19.9	>19.9	−1.70	4.10	14.90	18.60	>19.9	>19.9	−1.40	1.20	10.00	16.40	19.90	>19.9
	−1.64	−1.00	4.50	11.90	19.90	>19.9	>19.9	−0.90	3.80	13.90	18.40	>19.9	>19.9	–.80	.70	9.50	15.80	19.90	>19.9
	−1.28	−0.70	3.60	10.40	18.90	>19.9	>19.9	−0.90	3.10	12.10	17.30	>19.9	>19.9	–.10	.90	8.80	15.30	19.90	>19.9
	0.00	−0.40	0.60	2.00	10.50	17.20	>19.9	−0.10	−0.30	4.10	9.90	17.40	>19.9	.00	−1.30	1.20	7.60	14.10	>19.9
	1.28	−0.50	−1.80	−6.30	−7.00	−2.90	15.10	−0.20	−4.30	−8.50	−10.20	−4.30	16.10	−1.50	−6.30	−11.00	−12.40	−7.50	13.20
	1.64	−0.70	−2.80	−11.10	−14.80	−15.20	3.80	−0.50	−6.00	−12.30	−16.90	−15.60	5.40	−2.30	−7.90	−17.10	−21.40	−20.30	4.10
	1.96	−0.70	−3.70	−15.40	−21.20	−26.50	−12.90	−0.80	−7.30	−15.30	−23.70	−28.00	−13.30	−3.60	−9.90	−21.00	−28.30	−31.90	−16.30
CSA	−1.96	11.50	14.70	17.70	>19.9	>19.9	>19.90	10.90	17.10	19.10	19.60	>19.9	>19.9	12.80	13.70	16.00	19.90	>19.9	>19.9
	−1.64	7.70	11.40	15.80	>19.9	>19.9	>19.90	7.30	9.70	16.80	19.00	>19.9	>19.9	9.40	10.60	14.20	19.80	19.90	>19.9
	−1.28	2.40	6.50	12.50	19.90	>19.9	>19.90	1.10	4.80	13.40	18.50	>19.9	>19.9	3.10	5.50	11.80	16.00	19.90	>19.9
	0.00	−26.60	−22.20	−11.60	2.00	15.40	>19.90	−27.70	−23.50	−13.10	–.30	15.10	>19.9	−28.50	−26.40	−15.80	−1.40	12.00	>19.9
	1.28	−60.00	−58.30	−52.90	−44.30	−26.60	10.30	−59.20	−57.70	−52.30	−45.40	−28.80	12.80	−58.40	−57.60	−54.30	−46.80	−30.20	11.40
	1.64	−67.20	−65.30	−62.60	−56.50	−46.90	−6.10	−65.70	−64.50	−61.00	−56.00	−45.50	−6.00	−65.40	−65.20	−61.80	−57.30	−47.10	−5.60
	1.96	−71.40	−71.00	−68.70	−66.20	−59.30	−29.50	−69.60	−68.90	−67.30	−64.50	−57.60	−31.60	−69.80	−69.40	−67.90	−64.60	−59.00	−29.80
ANOVA	−1.96	−0.60	−0.80	0.20	−1.30	−0.30	0.80	−0.30	0.30	−0.70	0.20	0.10	0.10	2.00	1.00	1.70	1.90	2.80	2.60
	−1.64	−0.60	−0.80	0.20	−1.30	−0.30	0.80	−0.30	0.30	−0.70	0.20	0.10	0.10	2.00	1.00	1.70	1.90	2.80	2.60
	−1.28	−0.60	−0.80	0.20	−1.30	−0.30	0.80	−0.30	0.30	−0.70	0.20	0.10	0.10	2.00	1.00	1.70	1.90	2.80	2.60
	0.00	−0.60	−0.80	0.20	−1.30	−0.30	0.80	−0.30	0.30	−0.70	0.20	0.10	0.10	2.00	1.00	1.70	1.90	2.80	2.60
	1.28	−0.60	−0.80	0.20	−1.30	−0.30	0.80	−0.30	0.30	−0.70	0.20	0.10	0.10	2.00	1.00	1.70	1.90	2.80	2.60
	1.64	−0.60	−0.80	0.20	−1.30	−0.30	0.80	−0.30	0.30	−0.70	0.20	0.10	0.10	2.00	1.00	1.70	1.90	2.80	2.60
	1.96	−0.60	−0.80	0.20	−1.30	−0.30	0.80	−0.30	0.30	−0.70	0.20	0.10	0.10	2.00	1.00	1.70	1.90	2.80	2.60

Maximum increment is given as >19.9 since this would represent a conditional power in excess of 99.9%. Increments/decrements in power for ANOVA are due to random variation of the simulated dataset around the nominal 80% power.

The power of ANOVA is at its nominal level of 80% throughout, subject to some minor fluctuation from one simulation to the next (i.e. there are small fluctuations between the graphs in Figure 3). It is clear that for ANOVA, within any set of simulations (i.e. within any one graph in Figure 3), power is wholly unaffected by baseline imbalance, reflecting the fact that the statistical model for ANOVA has no term that represents such imbalance. It can be seen that if baseline imbalance is in the same direction as the treatment effect (indicated by positive values of *Z*), the power of both ANCOVA and CSA decreases with greater levels of imbalance, and CSA does so more markedly, especially at lower levels of *ZY* correlation. Thus, for a treatment effect of 0.2 and a *ZY* correlation of 0.1 (Figure 3 graph D), the power of CSA is as low as 9% if there were to be an extreme positive imbalance of 1.96. Conversely, when imbalance is in the opposite direction from the treatment effect, the power of both ANCOVA and CSA exceeds the nominal 80% power of ANOVA, and if *ZY* correlation is 0.7 or greater in these circumstances (Figure 3 graphs M to R), the superiority of ANCOVA and CSA is equivalent. If, however, *ZY* correlation is 0.3 or less in these circumstances, the power of CSA exceeds that of ANCOVA when negative baseline imbalance is most extreme (Figure 3 graphs D to I). If there is no baseline imbalance, the power of ANCOVA is either greater than or equal to that of ANOVA, whereas the power of CSA is superior to that of ANOVA at high correlations but inferior at low correlations. When *ZY* correlation is zero, ANCOVA has power approximately equivalent to that of ANOVA (Figure 3 graphs A to C).

## Discussion

This simulation study has examined the effect of baseline imbalance in an RCT on the bias and precision of estimates of treatment effect, and the power of a statistical test conditional on such imbalance. Although the statistical implications of baseline imbalance have previously been described, they have not hitherto been simultaneously quantified for these three analyses in relation to various combinations of levels of associated trial characteristics: effect size, degree of baseline-outcome (*ZY)* correlation and both magnitude and direction of baseline imbalance.

ANCOVA is known to produce unbiased estimates of treatment effect in the presence of baseline imbalance when groups are randomized [19,20]. ANOVA and CSA, however, produce biased estimates in such circumstances. For both ANOVA and CSA, the direction of bias is related to the direction of baseline imbalance, and bias is greatest when baseline imbalance, in either direction, is most pronounced. At a low *ZY* correlation, ANOVA exhibits less bias than CSA, but at a high *ZY* correlation the reverse is the case. In a situation in which ANOVA overestimates the unbiased treatment effect, CSA underestimates it, and vice versa. Both ANOVA and CSA show equal levels of bias (albeit in different directions) when the *ZY* correlation is 0.5. When *ZY* correlation is 0, estimates from ANCOVA and ANOVA are equivalent, as the absence of correlation means that the ANCOVA takes no account of imbalance and thereby reduces to ANOVA.

As regards precision, ANOVA and CSA yield less precise estimates than ANCOVA. ANOVA is progressively less precise than ANCOVA as *ZY* correlation increases; by contrast, CSA shows increasing precision as *ZY* correlation increases. CSA is less precise than ANOVA at *ZY* correlations below 0.5, but more precise at *ZY* correlations greater than 0.5, and both analyses present the same magnitude of associated standard error when the correlation is 0.5. In no situation do either CSA or ANOVA exceed the precision of ANCOVA.

The results for statistical power of the three analyses are not straightforward. The greater precision noted for ANCOVA might suggest that it would be unconditionally the most powerful analysis. Yet, as Figure 3 shows, whilst under some circumstances its power exceeds the nominal 80% power of ANOVA, under other circumstances ANOVA has greater power. This can be explained by the adjusted treatment effect derived through ANCOVA. When baseline imbalance is in the opposite direction from the treatment effect, ANCOVA corrects the resulting bias by producing an adjusted treatment effect that is larger than the nominal treatment effect, and ANCOVA therefore has greater power to detect this effect than ANOVA has to detect the nominal effect, at the same sample size. Correspondingly, when imbalance is in the same direction as the treatment effect, ANCOVA corrects the bias by adjusting the treatment effect downwards; its power to detect this effect is therefore less than that of ANOVA to detect the nominal treatment effect. However, when *ZY* correlation is 0 (Figure 3 graphs A to C), ANCOVA and ANOVA produce equivalent estimates of treatment effect, as noted earlier, and the difference in power therefore essentially disappears. This phenomenon also explains why baseline imbalance affects precision and power differently; precision is unaffected by imbalance but power reflects imbalance when it is calculated in relation to an adjusted treatment effect. When there is no imbalance, the adjusted treatment effect equals the nominal treatment effect and here ANCOVA is more powerful than ANOVA by virtue of its greater precision [18,31,32]. An important point to emphasize is that, in the presence of imbalance, nominal power is inappropriate due to the underlying bias in the estimation of the true treatment effect by ANOVA, which fails to address the baseline imbalance of the two treatment groups. As regards the analyses that accommodate baseline imbalance, ANCOVA is unconditionally more powerful than CSA, especially at lower *ZY* correlations [33].

The power of CSA shows a similar pattern to that of ANCOVA when ZY correlation is 0.7 or greater. At lower correlations, however, it demonstrates greater extremes of power than ANCOVA – higher than ANCOVA with imbalance in the opposite direction from the treatment effect and lower than ANCOVA with imbalance in the same direction. This indicates CSA’s over-correction of bias, in both directions, when *ZY* correlation is low; this stems from its failure to account for regression to the mean [24,34]. In the absence of imbalance, the power of CSA exceeds the nominal 80% power of ANOVA when *ZY* correlation is high, but is lower than that of ANOVA when *ZY* correlation is low. This reflects the relative precision of these two analyses conditional upon *ZY* correlation; CSA is the more precise at high correlations whereas ANOVA is the more precise a low correlations, as indicated by the ratios of standard errors in Table 2.

Relative to ANCOVA, the alternative analyses are thus liable to be either too conservative or too liberal [26]. It is clear therefore that the use of either ANOVA or CSA is inadvisable when baseline imbalance exists. Although all three methods are unbiased when there is no baseline imbalance, the likelihood is that in a clinical trial with several baseline covariates there will be some degree of imbalance across a number, if not all, of these variables. Similarly, the level of correlation between these covariates and the outcome variable is likely to be greater than zero (or possibly less than zero, though baseline values of the outcome variable are more likely to be positively than negatively correlated with post-treatment values). Moreover, ANCOVA is consistently the most precise method of analysis and hence delivers greatest efficiency in respect of testing against the null hypothesis and reducing the type II error. Our results concur with previous literature that emphasizes the advantages of covariate adjustment [3,8,9,12-16,24,35].

These simulations are based on imbalance in a single covariate. Where imbalance exists in a number of covariates, the degree of bias associated with either ANOVA or CSA will depend upon the combined effect of imbalances that may be in different directions, and upon the particular *ZY* correlations associated with each of these covariates. However, loss of precision (and hence of statistical power) through the use of ANOVA or CSA is likely to be greater with imbalance in multiple covariates than with imbalance in a single covariate, as there will normally be a greater proportion of variance in the outcome measure that is unaccounted for by either of these analyses.

Our results show the advantages of ANCOVA in reducing bias, increasing precision and providing appropriate power of statistical testing across a number of practical situations commonly seen in clinical trials. Several authors [2,34,36-39] argue that covariates should be selected a priori in terms of their prognostic importance, rather than on the basis of examining baseline imbalance in the trial data – even large imbalance is of little consequence in terms of bias if the covariate is not related to outcome. Moreover, the primary analysis in an RCT should be pre-specified [40,41]. Accordingly, our findings suggest that ANCOVA should be adopted as the analysis of choice, regardless of the magnitude of imbalance observed in the trial data. Consideration should also be given to achieving balance in important prognostic covariates at baseline in addition to subsequent statistical adjustment [42] – e.g. through stratified randomization or covariate-adaptive methods of allocation [11,43,44].

### Limitations

The conditions under which we have investigated the effect of baseline imbalance – in terms of magnitude of effect sizes, baseline imbalance and *ZY* correlation – are plausible and realistic, although the extremes of baseline imbalance examined will, reassuringly, be uncommon. Our findings are therefore readily transferable to specific real-life RCT scenarios. However, our findings assume equal allocation, and results may differ where this is not the case. Nor do our findings necessary generalize fully to trials where groups are not formed by randomization [45] or where outcomes are binary or time-to-event [28,42,46]. These results are also based on analyses whose assumptions were optimally satisfied through the simulation process, and are likely to differ in respect of real-life data that depart from such assumptions – e.g. a skewed outcome variable, or heterogeneous *ZY* regression coefficients between groups. Large trials will produce data that are robust to certain deviations in the assumptions underlying parametric analysis. Nonetheless, future work could usefully explore the impact of some of these deviations on the conclusions of the current study.

## Conclusion

In conclusion, ANCOVA should be the analysis of choice, a priori, for RCTs with a single post-treatment outcome measure previously measured at baseline; its superiority is particularly marked when baseline imbalance is present, but also – in terms of precision – when groups are balanced at baseline. We specifically caution against the use of ANOVA when the baseline-outcome correlation is (or is anticipated to be) moderate-to-large, and against CSA when it is (or is anticipated to be) small-to-moderate. Randomization generally leads to well-balanced groups, though non-systematic differences often arise across a number of covariates, and hence adjustment through ANCOVA is recommended to reduce risk of bias whilst also improving the precision of estimates and the power of the statistical test.

## Appendix

Simulation program in STATA. The prime identifies values that are specific to a particular simulation; i.e. r′ indicates r = 0.1, r = 0.3, r = 0.5, r = 0.7, r = 0.9; y′ indicates y = 0.2, y = 0.5, y = 0.8; z′ indicates standardized imbalance (the standard error of absolute imbalance multiplied by the appropriate standard normal deviate).

set seed

set obs n

[*defines number of observations (n) for the trial*]

g g = mod(_n,2)

[*defines two treatment groups – Control (0); Treatment(1)*]

g z = invnorm(uniform())*1

[*generates normally distributed baseline scores (z) with mean = 0 and SD = 1 and randomly assigns these to treatment groups*]

g r = r′

[*generates a predetermined correlation between baseline and post-treatment scores*]

g k = invnorm(uniform())*1

[*generates another normally distributed set of scores (k)*]

g y = z*r + k*(1−r^2)^.5

[*transforms k into an outcome score (y) that has a predetermined correlation with the baseline score (z)*]

replace z = z + g*z′

[*applies a predetermined direction-specific baseline imbalance to the treatment groups; with ‘z + g’, imbalance is in the same direction as the treatment effect, but with ‘z−g’ it is in the opposite direction*]

replace y = y + g*y′

[*creates a predetermined treatment effect*]

g c = y−z

[*generates change scores for the treatment groups*]

regress y g

[*performs analysis of variance*]

regress c g

[*performs change-score analysis*]

regress y g z

[*performs analysis of covariance*]

## Abbreviations

ANCOVA: Analysis of covariance; ANOVA: Analysis of variance; CSA: Change-score analysis; RCT: Randomized controlled trial.

## Competing interests

The authors have no competing interests.

## Authors’ contributions

JS and ML conceived the study. All authors designed the study. BEE planned and performed the simulations. All authors interpreted the data. All authors drafted the manuscript and approved the final version.

## Pre-publication history

The pre-publication history for this paper can be accessed here:

http://www.biomedcentral.com/1471-2288/14/49/prepub
